# Dihydromyricetin affects BDNF levels in the nervous system in rats with comorbid diabetic neuropathic pain and depression

**DOI:** 10.1038/s41598-019-51124-w

**Published:** 2019-10-10

**Authors:** Huixiang Ge, Shu Guan, Yulin Shen, Mengyun Sun, Yuanzhen Hao, Lingkun He, Lijuan Liu, Cancan Yin, Ruoyu Huang, Wei Xiong, Yun Gao

**Affiliations:** 1 Department of Physiology, Basic Medical College, Nanchang University, Nanchang, Jiangxi P.R. China; 2 Queen Mary College of grade 2016, Nanchang University, Nanchang, Jiangxi P.R. China; 3 Affiliated Stomatological Hospital of Nanchang University, Nanchang, Jiangxi P.R. China; 4 Jiangxi Provincial Key Laboratory of Autonomic Nervous Function and Disease, Nanchang, Jiangxi P.R. China; 5 Sport Biological Centre, China Institute of Sport Science, Beijing, P.R. China

**Keywords:** Diabetes complications, Neuropathic pain

## Abstract

Diabetic neuropathic pain (DNP) and depression (DP) are the common complications in patients with diabetes. The purpose of our research was to observe whether brain-derived neurotrophic factor (BDNF) levels and tropomyosin receptor kinase B (TrkB) in the nervous system have effects on rats with comorbid DNP and DP, and to determine whether dihydromyricetin (DHM) may influence BDNF/ TrkB pathway to mitigatethe comorbidity. The study showed that DHM treatment could attenuates pain and depressive behavior in DNP and DP combined rats. Compared with the control group, the expression level of BDNF/TrkB in the hippocampus of DNP + DP group were reduced, while the expression levels in the spinal cord and DRG were increased. However, after treatment with DHM, those changes were reversed. Compared with the control group, the level of IL-1β and TNF-α in the hippocampus, spinal cord and DRG in the DNP + DP group was significantly increased, and DHM treatment could reduce the increase. Thus our study indicated that DHM can relief symptoms of DNP and DP by suppressing the BDNF/TrkB pathway and the proinflammatory factor, and BDNF/TrkB pathway may be an effective target for treatment of comorbid DNP and DP.

## Introduction

Diabetes is a clinically chronic disease, and by 2045, the estimated worldwide incidence will rise to 693 million people^1^. There is a rising morbidity of diabetes in the world, and the diabetic population in China is the biggest^2^. A common symptom of diabetic complications is diabetic neuropathic pain (DNP), which has a great impact on the life of patients^3^. At present, the mechanism of DNP is not clear, and treatments have not been satisfactory. Therefore, it is especially important to seek more effective treatments.

Depression (DP) is a common public health disease that imposes an extremely serious financial burden on patients^4^. It has been estimated that the prevalence of depression worldwide is approximately 25%, and women are higher than men^5^. There is much evidence that there is a correlation between diabetes and depression^6,7^. Treatment of early depression can improve glycaemic control^8^. The risk of depression in patients with chronic pain is significantly higher than that in normal people, and the risk of chronic pain in patients with depression is also significantly higher than that in normal people^9^.

Some patients may have the comorbid conditions of DNP and DP, and the comorbidity brings more serious physical and mental effects to patients, and is more difficult to treat than only one disorder^10^. Patients with comorbid DNP and depression or anxiety have more healthcare costs than patients who are only suffering from DNP^11^. Therefore, it is extremely urgent to seek more effective treatments.

Brain-derived neurotrophic factor (BDNF) acts as a survival factor for neurons and is an important member of the neurotrophic family^12,13^. BDNF is a key signalling molecule in the microglia-neuron signalling pathway, and may be a therapeutic strategy for neuropathic pain treatment^14^. However, studies have shown that DP can reduce the expression of BDNF in hippocampus^12^. Some researchers had also confirmed that increased levels of BDNF in the hippocampus mediated the antidepressant-like effects of conventional antidepressants and ketamine, which makes BDNF an important target for depression^15^. Tropomyosin receptor kinase B (TrkB) is the high affinity receptor of BDNF. In comorbid DNP and DP conditions, the role of BDNF/TrkB pathway in the nervous system remains unclear and requires further research.

Dihydromyricetin (DHM) is extracted from a woody vine with the main active ingredient being flavonoids. DHM has many pharmacological effects such as anti-diabetic, anti-inflammatory, antioxidant and neuroprotective effects^16^. Our study aimed to observe the effects of BDNF/TrkB pathway in the nervous system on combined DNP and DP rats, and to determine whether DHM may influence BDNF/TrkB pathway to mitigate the comorbidity.

## Results

### Effect of DHM on thermal withdrawal latency (TWL) and mechanical withdrawal threshold (MWT) in rats with comorbid DNP and DP

**Figure 1:**
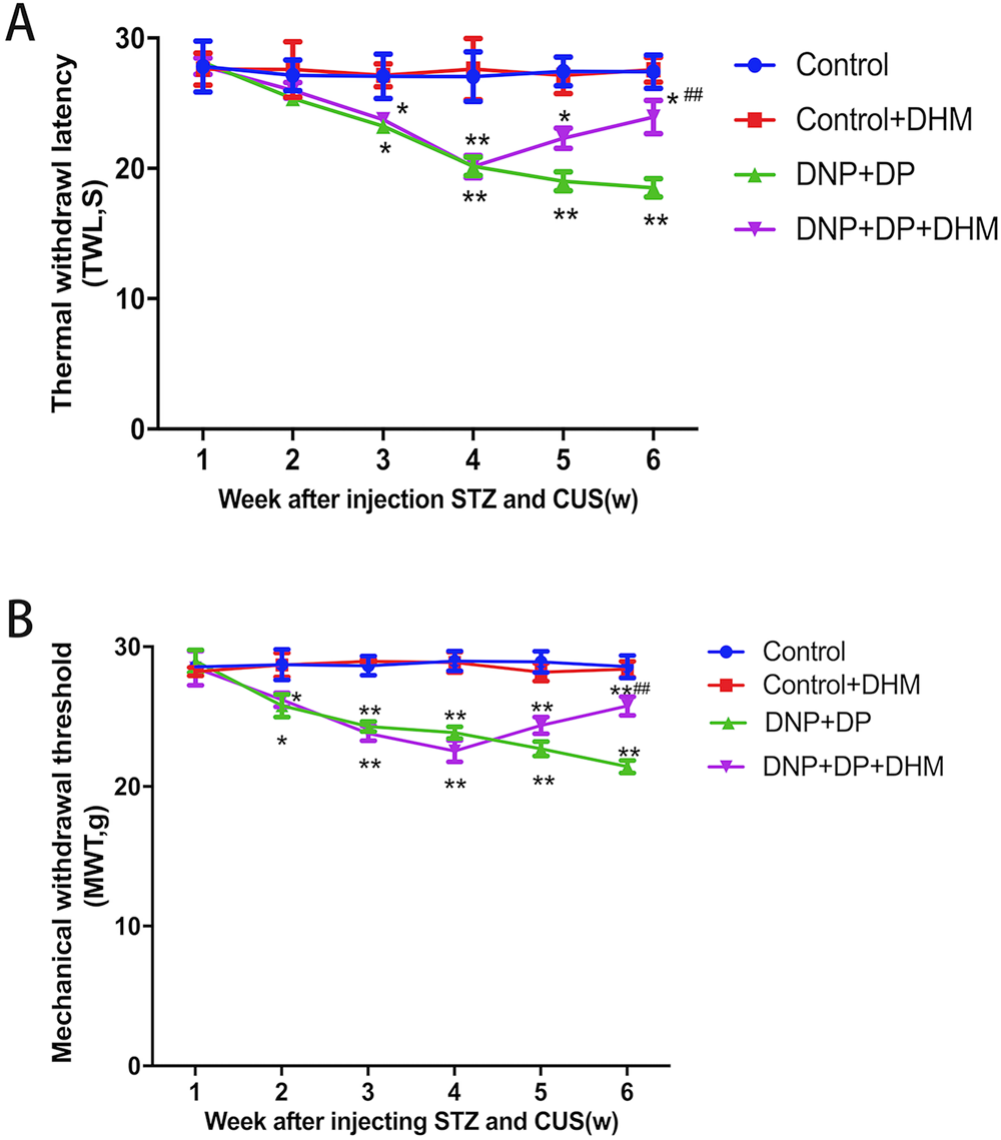
Effects of dihydromyricetin on the thermal withdrawal latency (TWL) (**A**) and mechanical withdrawal threshold (MWT) (**B**) of rats with comorbid DNP and DP. Values are the mean ± SEM of 6 observations. *p < 0.05 and **p < 0.01 *vs*. control group; ^##^p < 0.01 vs. DNP + DP group.

In this study, changes in pain related behaviours in rats were monitored by TWL and MWT. Four weeks after chronic unpredictable stress exposure, the TWL and MWT of rats in the DNP + DP group were significantly lower than in the Control group (p < 0.01). There were no differences in TWL and MWT between the control group and the control + DHM group (p > 0.05). After treatment with DHM for 2 weeks, TWL and MWT were markedly increased (p < 0.01). See Fig. 1.

The above data show that DHM treatment can increase TWL and MWT in DNP + DP rats, indicating that the neuropathic pain behaviours were mitigated by DHM.

### Effects of DHM on depressive behavior in rats with comorbid DNP and DP

**Figure 2:**
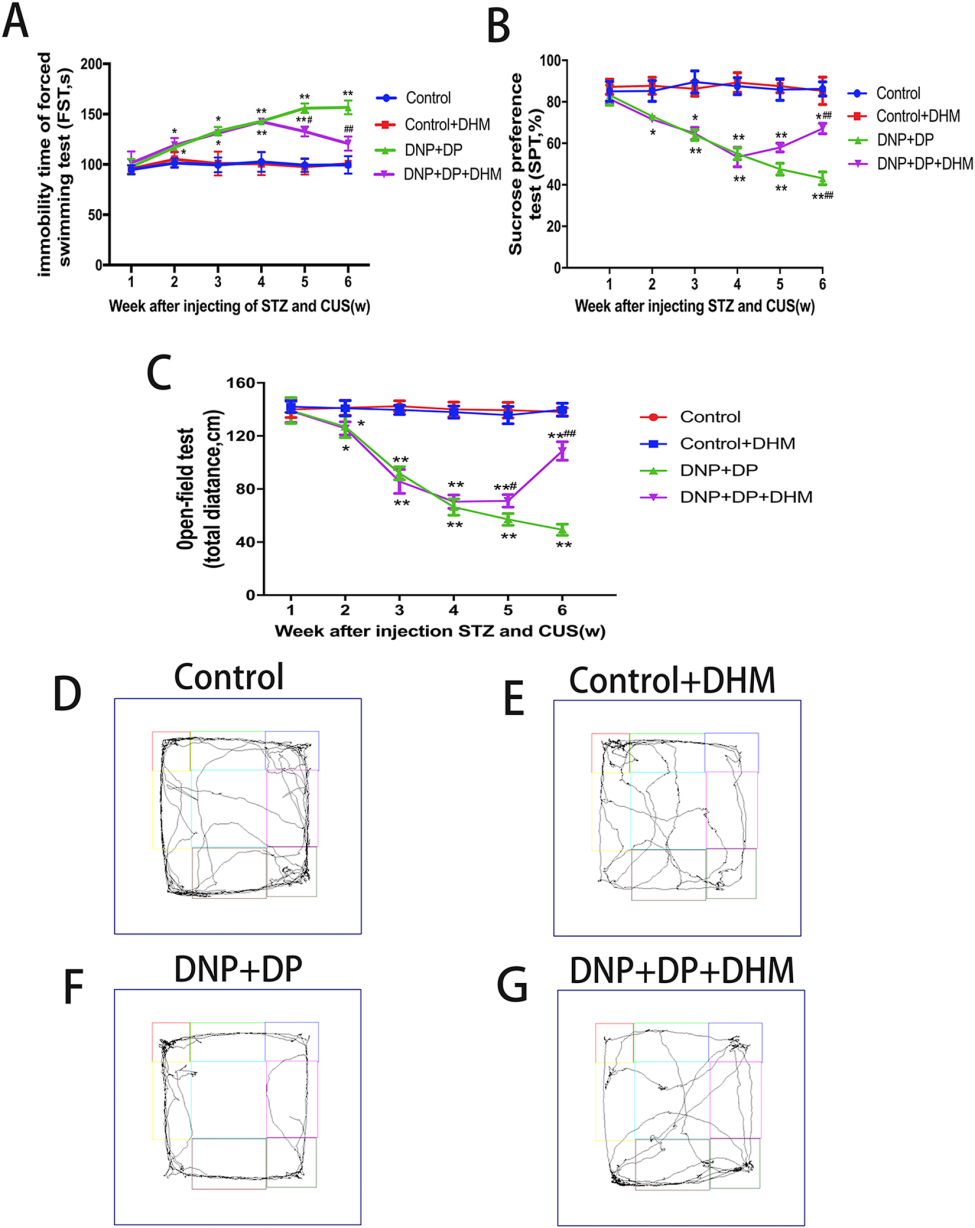
Effects of dihydromyricetin on the forced swimming test (FST) (**A**), sucrose preference test (SPT) (**B**) and open-field test (OFT) (**C**) in rats with comorbid DNP and DP. Figure D, E, F and G represented the total distance passed by the rat in each group recorded using a Canon camera model Powershot A610. Values are the mean ± SEM of 6 observations. *p < 0.05 and **p < 0.01 *vs*. control group; ^#^*p* < 0.05 and ^##^p < 0.01 *vs*. DNP + DP group.

After the DNP + DP model was successfully established, the levels of sucrose preference test (SPT) and open-field test (OFT) in DNP + DP rats were significantly lower than those in Control rats (p < 0.01), and the immobility time in the forced swimming test (FST) in DNP + DP rats was obviously higher than in the control rats (p < 0.01). After injection of DHM for two weeks, the levels of SPT and OFT in the DNP + DP + DHM group were obviously higher than those in the DNP + DP group (p < 0.01).Furthermore, the immobility time of FST in the DNP + DP + DHM group was lower than that in the DNP + DP group (P < 0.01). See Fig. 2.

The above data showed that the depression-related behaviours were relieved by DHM.

### Effects of DHM on the expression levels of BDNF and TrkB in rats with comorbid DNP and DP

**Figure 3:**
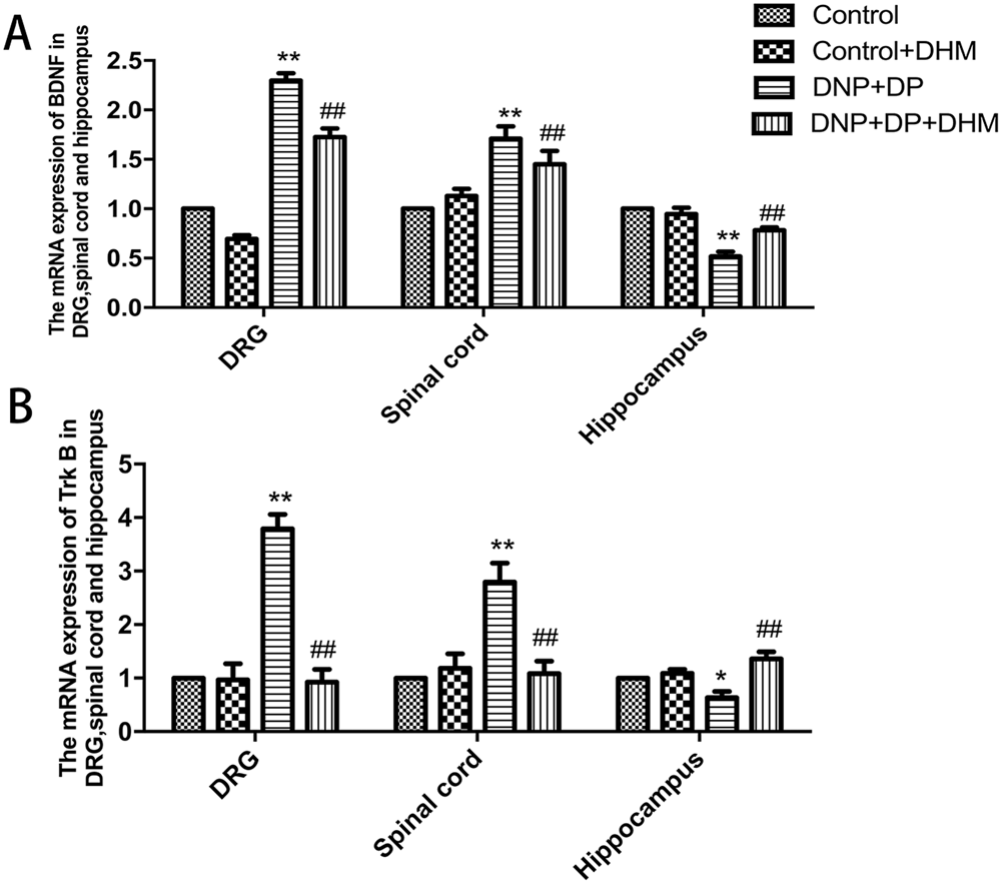
Effects of dihydromyricetin on the expression of BDNF (**A**) and TrkB (**B**) mRNA in the DRGs, spinal cord and hippocampus of rats with comorbid DNP and DP as detected by qRT-PCR. Values are the mean ± SEM of 6 observations. *p < 0.05 and **p < 0.01 *vs*. control group; ^##^*p* < 0.01 *vs*. DNP + DP group.

**Figure 4:**
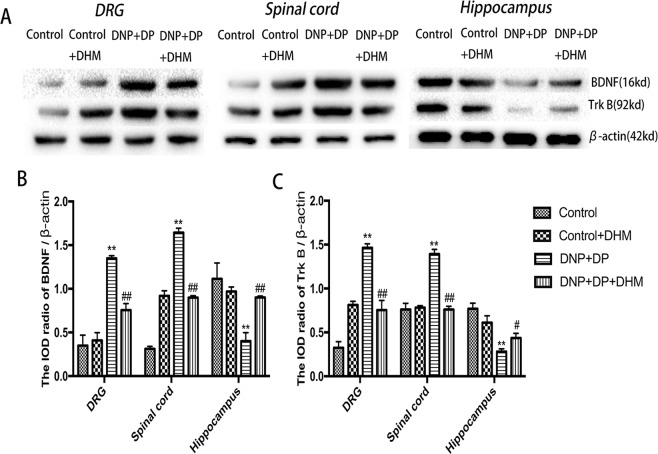
Effects of dihydromyricetin on the expression of BDNF (**A**,**B**) and TrkB (**A**,**C**) protein in the DRGs, spinal cord and hippocampus of DNP + DP rats as detected by Western blotting. Values are the mean ± SEM of 6 observations. *p < 0.05 and **p < 0.01 *vs*. control group; ^#^*p* < 0.05 and ^##^*p* < 0.01 *vs*. DNP + DP group.

We used quantitative real-time PCR and Western blotting to detect mRNA and protein expression levels of BDNF and TrkB in the rats. The results showed that in DRG and spinal cord tissue, the mRNA and protein expression levels of BDNF and TrkB were higher in the DNP + DP group than in the Control group, but in thehippocampus, the expression levels of BDNF and TrkB in the DNP + DP group were lower than in the Control group (p < 0.01). DHM treatment can significantly reverse these changes (p < 0.01). See Figs 3 and 4.

**Figure 5:**
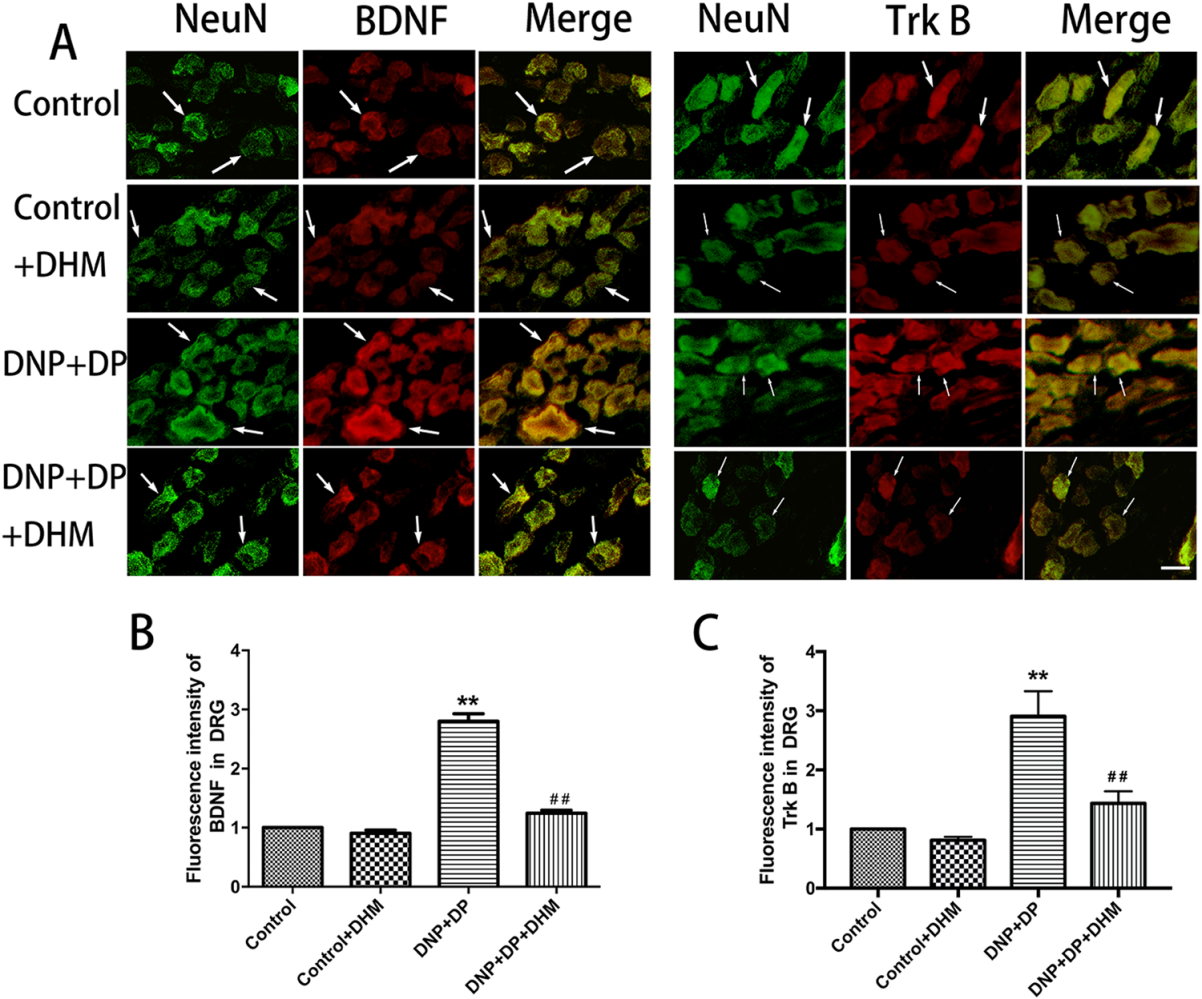
Co-expression of NeuN-BDNF (**A**,**B**) and NeuN-TrkB (**A**,**C**) in the DRG was detected with double-labelled immunofluorescence. Values are the mean ± SEM of 3 observations. **p < 0.01 *vs*. control group; ^##^*p* < 0.01 *vs*. DNP + DP group. Scale bar: 20 μm.

**Figure 6:**
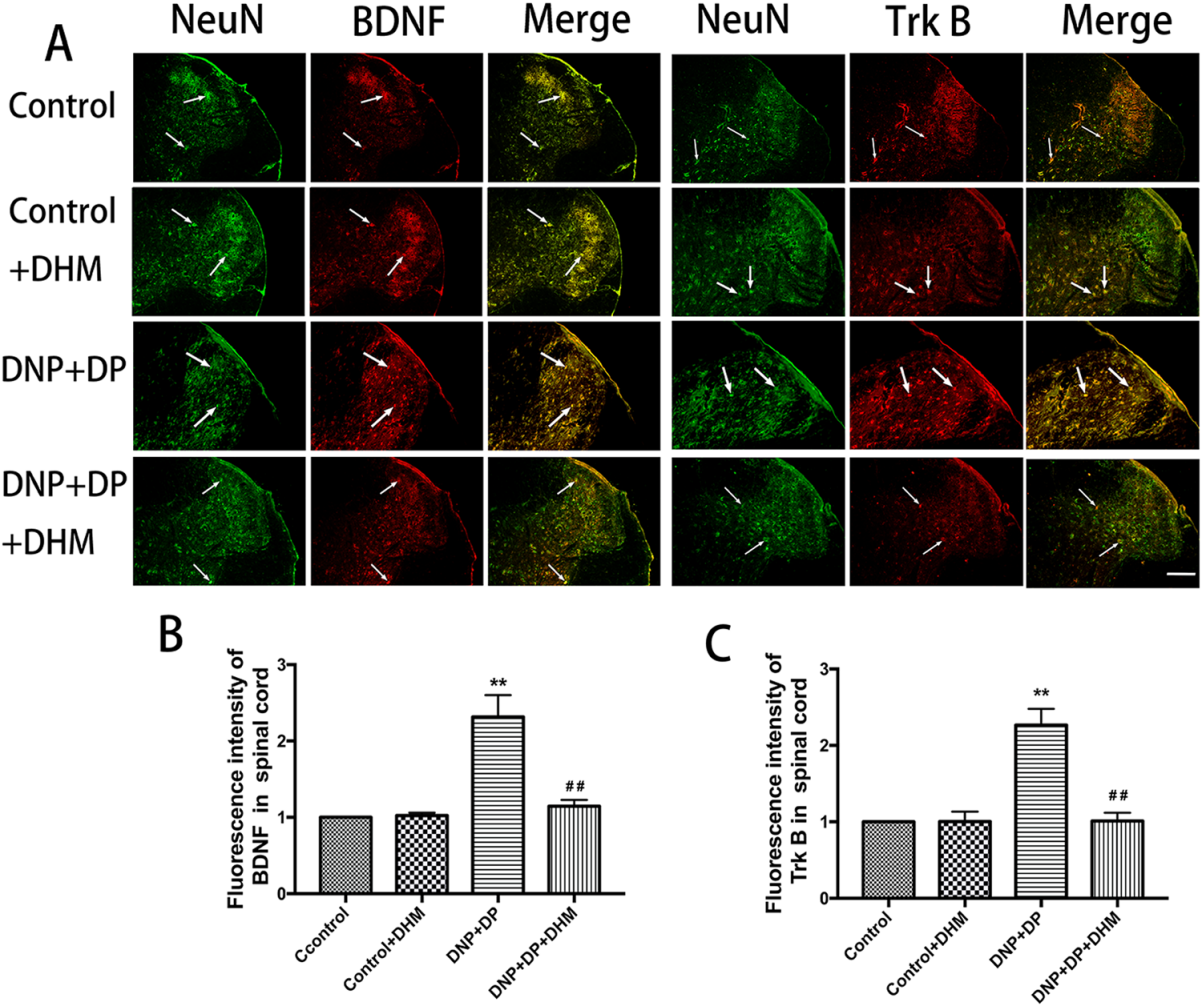
Co-expression of NeuN-BDNF (**A**,**B**) and NeuN-TrkB (**A**,**C**) in the spinal cord was detected with double-labelled immunofluorescence. Values are the mean ± SEM of 3 observations. **p < 0.01 *vs*. control group; ^##^*p* < 0.01 *vs*. DNP + DP group. Scale bar: 100 μm.

**Figure 7:**
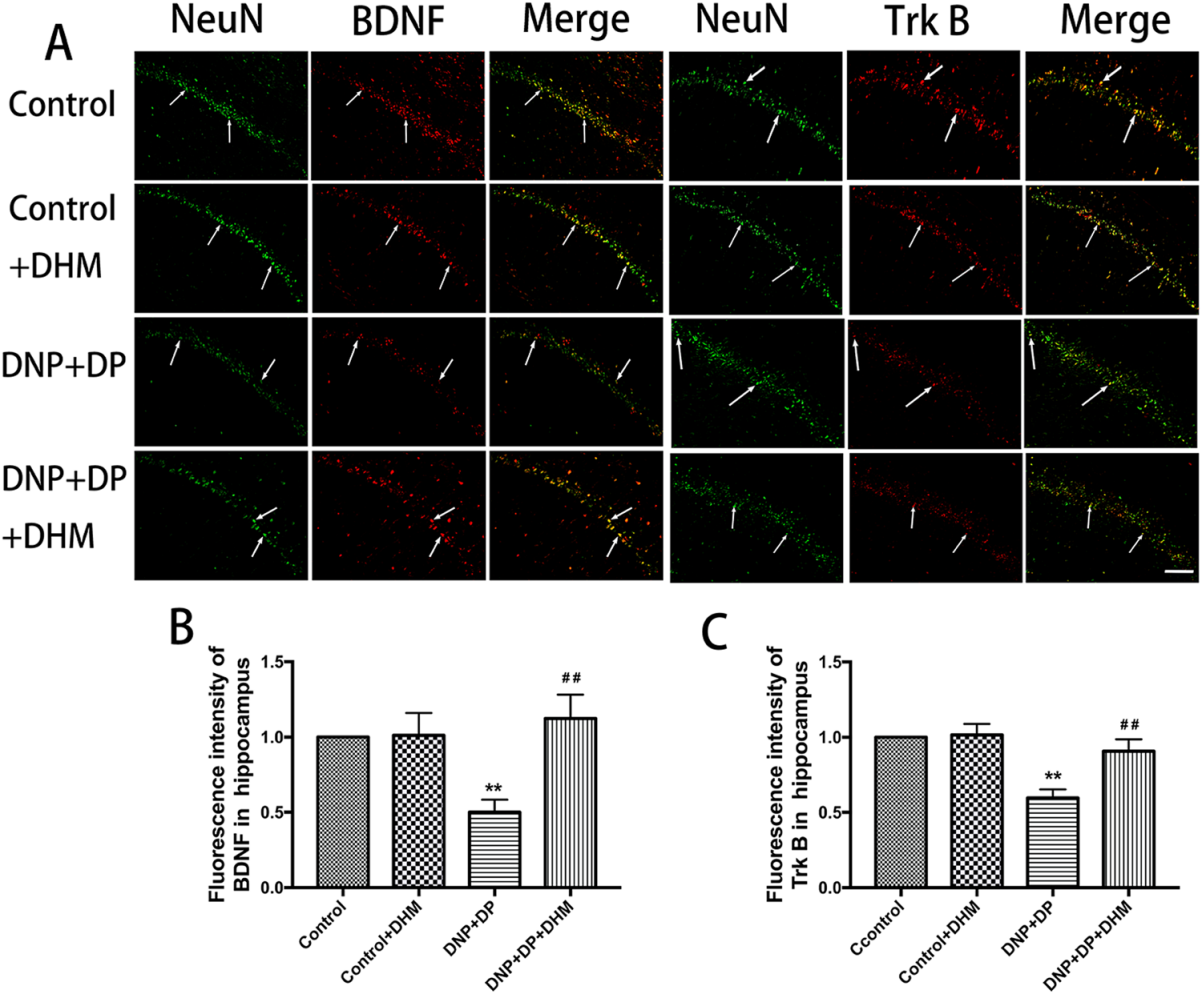
Co-expression of NeuN-BDNF (**A**,**B**) and NeuN-TrkB (**A**,**C**) in the hippocampus was detected with double-labelled immunofluorescence. Values are the mean ± SEM of 3 observations. **p < 0.01 *vs*. control group; ^##^*p* < 0.01 *vs*. DNP + DP group. Scale bar: 200 μm.

We also used immunofluorescence double-labelling to detect the immunoreactivity of BDNF and TrkB in the hippocampus, spinal cord and DRG. NeuN is a marker of neurons. The results confirmed that the co-expression of BDNF-NeuN and TrkB-NeuN in these tissues, and BDNF and TrkB were mainly expressed in neurons in DRG. The results also showed that the co-expression of BDNF-NeuN and TrkB-NeuN in the spinal cord and DRG were higher inthe DNP + DP groupthan in the control group, and treatment with DHM could block this increase (p < 0.01). In the hippocampus, the co-expression of the BDNF-NeuN and TrkB-NeuN was lower in the DNP + DP group than that in the control group, and DHM treatment increased the change (p < 0.01). See Figs 5, 6 and 7.

The above data indicated that DHM may reverse the expression of BDNF and TrkB receptors in the hippocampus, spinal cord and DRG of DNP and DP comorbid rats.

### Effects of DHM on the levels of IL-1β and TNF-α in rats with comorbid DNP and DP

**Figure 8:**
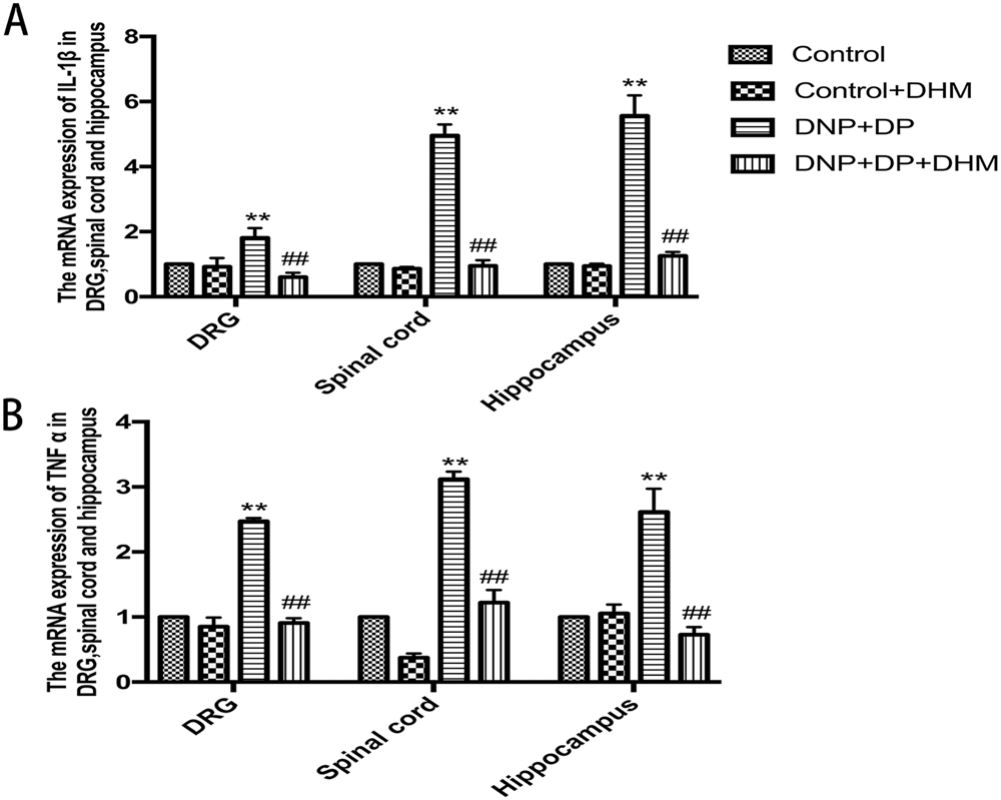
Effects of DHM on the expression levels of IL-1β (**A**) and TNF-α (**B**) mRNA in the DRG, spinal cord and hippocampus of rats with comorbid DNP and DP as detected by qRT-PCR. Values are the mean ± SEM of 6 observations. *p < 0.05 and **p < 0.01 *vs*. control group; ^##^*p* < 0.01 *vs*. DNP + DP group.

The mRNA expression levels of IL-1β and TNF-α in the hippocampus, spinal cord and DRG were detected by qPCR. The results showed that the mRNA expression levels of IL-1β and TNF-αwere higher in the DNP + DP group than those in the Control group. However, the expressions of IL-1β and TNF-α mRNA were lower in the DNP + DP + DHM group than those in the DNP + DP group (p < 0.01). See Fig. 8.

**Figure 9:**
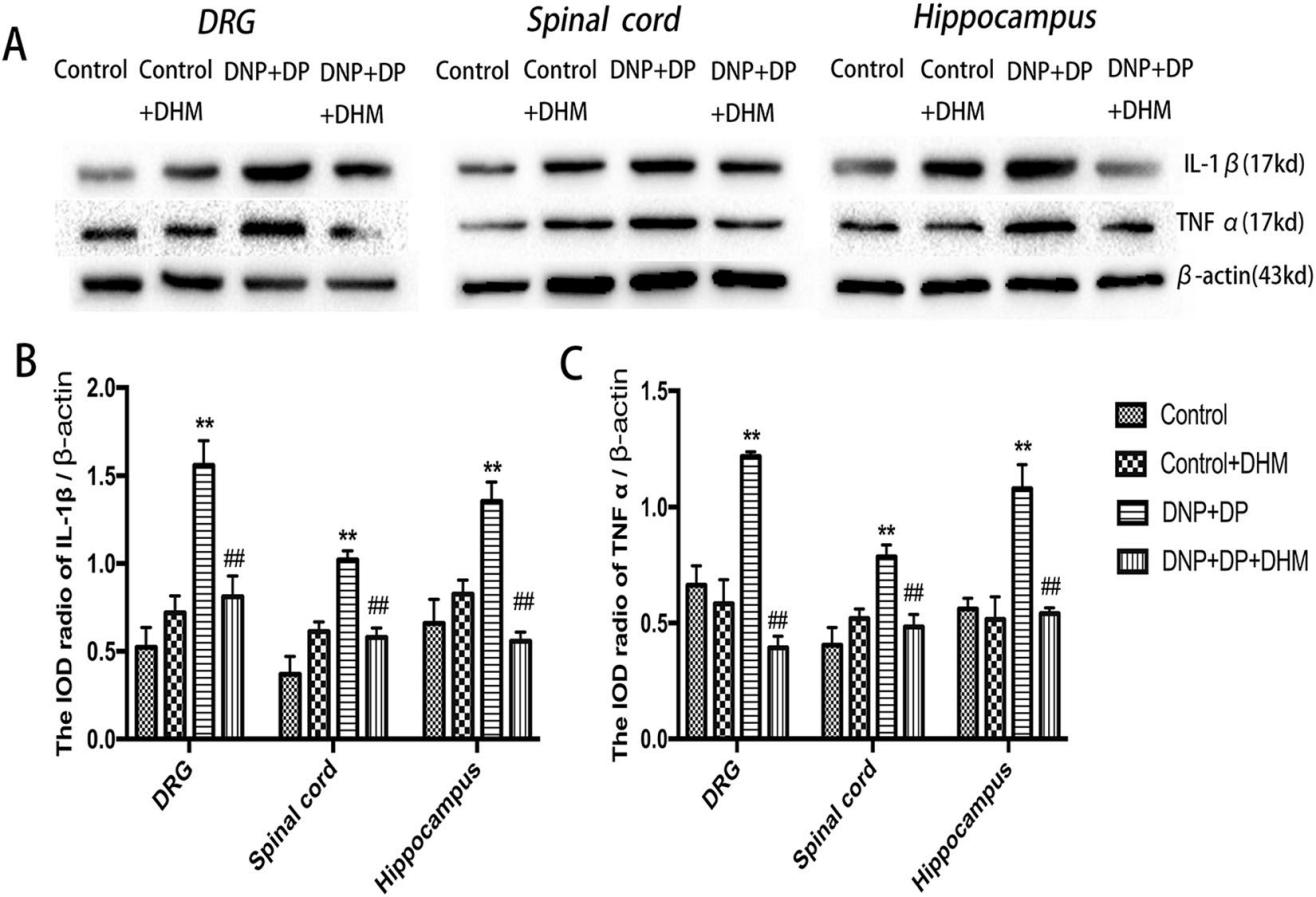
Effects of DHM on the expression levels of IL-1β (**A**,**B**) and TNF-α (**A**,**C**) protein in the DRG, spinal cord and hippocampus of DNP + DP rats as detected by Western blotting. Values are the mean ± SEM of 6 observations. *p < 0.05 and **p < 0.01 *vs*. control group; ^#^*p* < 0.05 and ^##^*p* < 0.01 *vs*. DNP + DP group.

In addition, we also detected the IL-1β and TNF-α level in each group using Western blotting. The IL-1β and TNF-α protein levels were higher in the DNP + DP group than those in the Control group. But the DHM treatment could down-regulate the increased change to normal level (p < 0.01). See Fig. 9.

## Discussion

Clinically, diabetes is a high-risk chronic disease. Diabetes patients often have multiple complications. DNP and DP are twoof themost common complications in diabetes patients. The two diseases can sometimes co-occur, which greatly impacts the quality of life of patients^17,18^. At present the mechanism is still unclear and traditional pharmacotherapies for chronic pain and depression cannot meet the needs of patients with comorbid DNP + DP^17,19^. Therefore, it is urgent to elucidate the mechanism underlying this comorbidity and develop new drugs to treat patients with the DNP + DP comorbidity more effectively.

In our study, a rat model of DNP and DP was established by a high-sugar and high-fat diet, injecting STZ, and exposing them to chronic unpredictable stress^20^. Meanwhile, we measured responses in several ways per week to verify that rats had both symptoms. The significantly reduced values for the TWL, MWT, SP, and OFT distance, as well as the significantly increased FST values, confirmed the successful generation of the DNP + DP model.DHM is a natural flavonoid in *Ampelopsis grossedentata*, such as vine tea, which has various functions such as anti-inflammatory and anti-tumor effects^16,21,22^. The results of these behavioural tests showed that DHM treatment can reverse these changes, indicating that DHM therapy can mitigate the symptoms of the comorbidity.

BDNF is widely present in the peripheral and central nervous systems and plays an important role in maintaining pain, depression, anxiety and memory-related functions^23^. BDNF has a protective effect in Parkinson’s disease^24^, Alzheimer’s disease^25^, metabolic syndrome^26^ and depression^27^, however,BDNF has facilitated effects on the transmission of neuropathic pain^28^. The regulation of BDNF concentrations is directly related todepressionaetiology;for example, the upregulation of BDNF causes an antidepressant-like effect and the downregulation of BDNF accounts for depression^29,30^. Meanwhile many studies have confirmed that BDNF/TrkB is involved in neuropathic pain progression, reducing BDNF/TrkB signalling in the spinal dorsal horn produced anti-neuropathic pain effects^31–33^. During the comorbidity of DNP and DP, our previous study found a decrease in the expression of BDNF in the hippocampus^20^. However, at present the effects of BDNF/TrkB signaling in the nervous system are not completely understandin the context of comorbid DNP and DP. Thus, we assessed the expression of BDNF/TrkB in the hippocampus, spinal cord and DRG of L4/L5. The results of qPCR and Western blotting tests indicated that the level of BDNF/TrkB in the hippocampus was lower in comorbid DNP and DP rats than in the control group, but the results in the DRG and spinal cord were opposite to those in the hippocampus. DHM treatment can block these changes. We also used immunofluorescence double-labelling to detect the immunoreactivity of BDNF and TrkB in the hippocampus, spinal cord and DRG, respectively. Our study found there were co-expressions of BDNF-NeuN and TrkB-NeuN in these tissues. The co-expression of the BDNF-NeuN and TrkB-NeuN in the spinal cord and DRG were higher in the DNP + DP group than in the control group, but it was lower in the hippocampus. DHM treatment can also reverse these changes. Thus we hypothesized that there are different mechanisms in the hippocampus, spinal cord and DRG for BDNF/TrkBsignalling during comorbid DNP and DP, and DHM can relieve the comorbidity by normalizing the changes of BDNF/TrkBsignalling in the nervous system.

Studies have confirmed that chronic pain, depression behavior and neuroinflammation are closely related^34,35^. Inhibiting TNF-α can prevent stress-induced memory disorder by maintaining BDNF levels of hippocampus in the CUS rat model of depression^36^. Lithium may be a neuroprotective agent against the induced toxicity of methylphenidate by suppressing hippocampal BDNF, IL-1β and TNF-α^37^. Our previous study had founded that the hippocampal IL-1β and TNF-α levels of rats with DNP and DP were significantly higher than control rats^20^. In this study the results were similar to the previous research. DHM treatment can block the increased inflammatory factors. We speculate that the high inflammatory factor levels in the hippocampus will decrease BDNF/TrkB expression to develop the depression, but in the spinal cord and DRG, the high inflammatory factor levels will increase BDNF/TrkB expression to facilitate the transmission of pain. However, the exact underlying mechanism still needsadditional exploration. We provide support that DHM has antiinflammation effects, can decrease thelevels of IL-1β and TNF-αin the hippocampus,spinal cord and DRGof model rats, and then influence BDNF/TrkB expression to mitigate the comorbidity of DNP and DP.

## Conclusion

In conclusion, DHM can alleviate the symptoms of diabetic neuropathic pain and depression by regulating the level of BDNF expression in the nervous system, and its mechanism may involve the inhibition of the release of the inflammatory factors IL-1β and TNF-α.DHM is expected to become a new drug for the treatment of the comorbidity of DNP and DP.

## Materials and Methods

### Animals and treatments

Male Sprague Dawley (SD) rats(180–220 g) were supplied by the Centre of Laboratory Animal Science of Jiangxi University of Traditional Chinese Medicine. The Animal Care and Ethics Committee of Nanchang University approved the procedures of this research. The IASP’s ethical guidelines for the study of pain in animals were followed. Five SD rats were housed in each cage at a temperature of 25 °C and a humidity of 60%, and provided freely accessible food and water.

The first week before the experiment started,SD rats were fed aregular diet. Then, for the next 4 weeks, rats were fed a high-glucose-high-fat diet. After a 12-h fast on the weekend of week 4, they were given an intraperitoneal injection of streptozotocin(STZ) (35 mg/kg)^20,38,39^.

**Figure 10:**
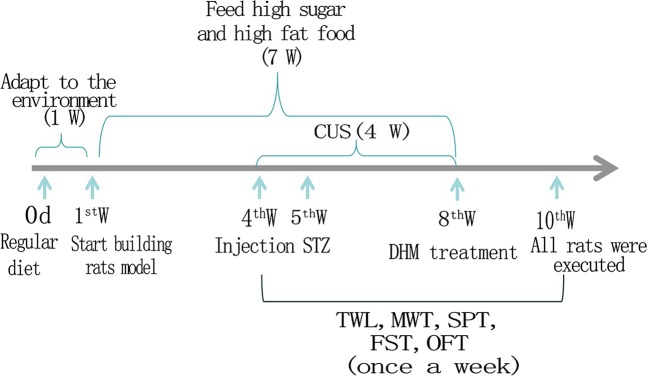
Timeline of the model establishment and sample collection.

Rats were randomly tested and those with blood glucose concentrations above 16.7 mmol/L were selected as type 2 diabetes mellitus rats. Then within four weeks after STZ injection, rats were exposed to a chronic unpredictable stress (CUS) procedure.During this period, the thermal withdrawal latency (TWL), mechanical withdrawal threshold (MWT), the sucrose preference test (SPT), the immobility time of forced swimming test (FST) and the open-field test (OFT) were monitored once a week to select the rats with comorbid diabetic neuropathic pain and depression. The CUS procedureis a classic animal model of depression^40–42^, incorporating reversed light/dark cycle, food deprivation (24 h), water deprivation (24 h), tail pinch (1 min), swimming in cold water (4 °C, 5 min), swimming in hot water (45 °C, 5 min), and no stressor. These seven kinds of conditions were administered daily at random for five weeks. Sixty male SD rats were stochastically divided into 4 groups as follows: (1) control group (Control); (2) control + dihydromyricetin group (Control + DHM); (3) diabetic neuropathic pain and depression group (DNP + DP); (4) dihydromyricetin treatment group (DNP + DP + DHM). Rats in the Control + DHM and DNP + DP + DHM groups were injected intraperitoneally with DHM for 2 weeks at a dose of 30 mg/kg^43^. Figure 10 shows the timeline for the establishment of an animal model. STZ was purchased from Solarbio. DHM ((2 R,3 R)-3,5,7-trihydroxy-2-(3,4,5-trihydroxyphenyl) benzoin-4-one; CAS No. 27200-12-0; HPLC purity ≥ 98%) was provided by Zelang Co., Nanjing, China.

### Mechanical withdrawal threshold (MWT)

The MWT was evaluated by a paw withdrawal response to mechanical stimulation by BME-404 electromechanical stimulator (Institute of Biomedical Engineering, Chinese Academy of Medical Sciences)^44,45^. The test needle’s face diameter was 0.6 mm, the pressure measurement range was from 0.1 g to 50 g, and the pressure measurement resolution was 0.05 g. Rats rested on the wire mesh for at least 30 min as an adaptation time prior to the test. The same observer used the test needle to touch the left hind paw of the rat (between the third and fourth metatarsal) and applied force until the paw tried to lift. The MWT was recorded via the computer and expressed in grams. The stimulation interval was 2 min. The average of the three consecutivestable measurementswas obtained.

### Thermal withdrawal latency (TWL)

The TWL was evaluated by radiant heat and the BME-410C Thermal Paw Stimulation System (Institute of Biomedical Engineering, Chinese Academy of Medical Sciences) to obtain the retraction latency after thermal stimulation in the rats^44,45^. Prior to testing, each rat was placed in a transparent box on the glass surface for 30 min.The test began with a beam of radiant heat stimulating the underside of the midfoot surface of the hind paw and activation of the timer to begin the trial. The cutoff time for thermal stimulation was 30 s. When the rat lifts the paw and disconnects the light source, the timer ends. The time displayed on the timer was recorded as TWL and expressed in seconds.Finally, the average of the three stable data points was taken and the test was initiated at 2 min intervals.

### Sucrose preference test (SPT)

Before the test, rats were fasted for 24 h and then placed individually in separate cages. Two identical water bottles, one containing 100 ml of 1% sucrose in water and another containing 100 ml pure water, were placed in all cages at the same time. SP was evaluated by measuring the levels of sugar-water and pure-water consumption in one hour^20,46^. The SP rate was calculated as the ratio of sugar water/total liquid consumption x 100%.

### Forced swimming test (FST)

Rats were placed in an 80-cm-high glass cylinder with a 40-cm inner diameter. The water temperature was approximately 20 °C, and the water depth was 30 cm. The immobility time of FST of rats in the water, i.e., when rats stopped struggling and floated in a fixed shape, was recorded for 5 min and expressed in seconds^20,47^.

### Open-field test (OFT)

The rats were placed in a dark environment for 30 minutes before the test. Then, they were placed in a black box that measured 40 × 60 × 50 cm. Each rat was placed gently in the middle of the box, and the animal’s behavior was recorded using a Canon Powershot A610 camera (Canon Co. local distributer, Tehran, Iran) during a 5-min session. The recorded videos were analysed and processed using MATLAB (Mathworks Co., Massachusetts, USA) to determine the total distance travelled, expressed in centimetres^20,48^. The apparatus was cleaned with a 10% ethanol solution before the next animal was introduced into the box.

### Quantitative real-time PCR (qPCR)

Rats were anaesthetized by i.p. injection of 10% chloral hydrate (Batch No: 050101; Shanghai Xingya Medical Company, China). DRGs, the spinal cord at the level of L4-L5 vertebrae, and hippocampus were isolated immediately after sacrifice from rats in the different groups, flushed with ice-cold PBS, and stored in RNA Store solution at −20 °C until use. All instruments were treated with DEPC before use.

Total RNA was separated from tissue sample using the TRIzol Total RNA Reagent(Beijing TransGen Biotech Co.). Total RNA (2 µg) was used to synthesise complementary DNA (cDNA) by the RevertAid™ HMinus First Strand cDNA Synthesis Kit. Applied Primer Express 3.0 Software to designed the primer sequences, as follows:

β-actin forward 5′-TAAAGACCTCTATGCCAACA -3′ and reverse 3′-CACGATGGAGGGGCCGGACTCATC -5′;

TrkB forward 5′-TGGAGGAAGGGAAGTCTGTG -3′ and reverse 3′-AGTGGTGGTCTGAGGTTGGA -5′;

BDNF forward 5′-CCTCTGCTCTTTCTGCTGGA -3′ and reverse 3′-GCTGTGACCCACTCGCTAAT -5′;

IL-1β forward 5′-CCTATGTCTTGCCCGTGGAG-3′ and reverse 3′-CACACACTAGCAGGTCGTCA-5′;

TNF-α forward5′-CACGTCGTAGCAAACCACCAA-3′ and reverse 3′-GTTGGTTGTCTTTGAGATCCAT-5′.

QPCR was performed using the SYBR® Green Master Mix in the ABI PRISM® 7500 Sequence Detection System (Applied Biosystems Inc., Foster City, CA). The expression of each gene was quantified using the ΔΔCT method, with CT as the threshold cycle^20^. The relative levels of target genes were obtained by the software within the ABI7500 PCR instrument.

### Western blotting experiment

After rats were anaesthetized, the hippocampus, DRGs and spinal cord at L4-L5 were separated and flushed with ice-cold PBS and stored at −20 °C. Tissue was positioned in a spherical portion of a 2 ml homogenizer, and moderate RIPA lysis buffer(Beyotime Biotechnology, Shanghai, China) was added to homogenize the sample. Tissue was ground for 30 min on ice and centrifuged at 4 °C at 12000 rpm for 10 min. The supernatants were collected, diluted with 6x loading buffer, and heated to 95 °C for 10 min. The protein concentration was calculated with the BCA Protein Assay Kit, and samples were kept at −20 °C until use. Proteins in samples from each group (20 μg) were separated by 12% SDS-polyacrylamide gel electrophoresis, using a Bio-Rad electrophoresis device, and transferred onto polyvinylidene fluoride (PVDF) membranes. The membranes were incubated with 5% non-fat dry milk prepared with 1X TBST for 2 h at normal temperature. Next, PVDF membranes were incubated with rabbit antibodies against TNF-α (1:800 concentration, Abcam, USA), IL-1β (1:800 concentration, Abcam, USA), BDNF (1:500 concentration, Abcam, USA), β-actin (1:1000 concentration, Beijing Zhongshan Biotech Co., China), and TrkB (1:1000 concentration, Abcam, USA) at 4 °C overnight. The membranes were washed 3 times with 1x TBST every 10 min. The secondary antibody (goat anti-rabbit IgG, 1:2000 concentration, Beijing Zhongshan Biotech Co., China) was diluted with blocking buffer and incubated at room temperature for 2 h. The PVDF membranes were washed every 10 min 3 times. The labelled proteins were then visualized with enhanced chemiluminescence on a Bio-Rad system. Band intensities were quantified using Image-Pro Plus software, and the intensities of target proteins were normalized against the respective β-actin internal control^20^.

### Double-labelled immunofluorescence

Rats were anaesthetized with 10% chloral hydrate and were transcardially perfused with 4% paraformaldehyde (PFA). The hippocampus,spinal cord and DRG were removed and placed in 4% PFA solution at 4 °C. Dehydration was carried out by immersing tissue for 24 h at 4 °C in 30% sucrose solutions (in 4% PFA), and the solutions were renewed every 8 h. DRG were cut into 8-µm-thick slices by a cryostat microtome (Leica). The hippocampus tissuewascut into 10µmslices and the spinal cord tissue was cut into 12 µm slices. Tissue sections were dried at 37 °C for 2 h, and then stored at −20 °C. Before staining, sections were balanced at room temperature for 30 min, rinsed with 0.01 M PBS for 5 min x 3 times, incubated with 0.3% Triton X-100, and washed again with PBS for 5 min x 3 times. Then, slices were incubated in 10% goat serum for 1 h at 37 °C, followed by incubation with the diluted antibodies (rabbit anti-BDNF, 1:500, Abcam, USA; rabbit anti-TrkB, 1:500, Abcam, USA; mouse anti-GFAP, 1:200, Millipore) overnight at 4 °C. Secondary antibodies (Goat anti mouse 1:800, Abcam, USA; Goat anti rabbit 1:800,Abcam, USA) were added for incubation at 37 °C for 1 h. Sections were washed for 5 min x 3 times with PBS, sealed with antifade solution, and imaged using a fluorescence microscope (Olympus, Tokyo, Japan). The immunofluorescence intensity was analysed using Image-Pro Plus6.0 software^20^.

### Statistical analysis

Statistical analyses were performed using SPSS21 software. Data were analysed with a one-way analysis of variance (ANOVA) followed by LSD post-hoc test for multiple comparisons. The experimental results are expressed as the mean ± standard error of the mean and were considered significant at p < 0.05.

### Ethical approval

The Animal Care and Ethics Committee of Nanchang University approved the procedures of this research. The IASP’s ethical guidelines for the study of pain in animals were followed.

## Supplementary information

Supplementary information

## Data Availability

The datasets generated during and/or analysed during the current study are available from the corresponding author on reasonable request.
