# Supplementary material for: Dihydromyricetin affects BDNF levels in the nervous system in rats with comorbid diabetic neuropathic pain and depression
Source: Sci Rep. 2019 Oct 10;9:14619. doi: 10.1038/s41598-019-51124-w (PMC6787069; doi:10.1038/s41598-019-51124-w)
Supplement: Supplementary file 1 — Supplementary information [file 41598_2019_51124_MOESM1_ESM.pdf]

Supplementary information

For the gels and blots, we only selected a portion of the results of the Western blot that represents this experiment. And in the relevant experimental research, the expression of this result is also adopted.

Title page

1.Title: Dihydromyricetin affects BDNF levels in the nervous system in rats with comorbid diabetic neuropathic pain and depression

2. Running Title: Dihydromyricetin and comorbid diabetic neuropathic pain and depression

3. All authors and institution:

[1]Huixiang Ge, [1]Shu Guan, [1,5]Yulin Shen, [1]Mengyun Sun, [2]Yuanzhen Hao, [3]Lingkun He, [3]Lijuan Liu, [3]Cancan Yin, [3]Ruoyu Huang, [3]Wei Xiong, [1,4,*]Yun Gao

[1]Department of Physiology, Basic Medical College, Nanchang University, Nanchang, Jiangxi, P.R. China;

[2]Queen Mary College of grade 2016, Nanchang University, Nanchang, Jiangxi, P.R. China;

[3]Affiliated Stomatological Hospital of Nanchang University, Nanchang, Jiangxi, P.R. China;

[4]Jiangxi Provincial Key Laboratory of autonomic nervous function and disease, Nanchang, Jiangxi, P.R. China

[5]Sport Biological Centre, China Institute of Sport Science, Beijing, P.R. China

4. Corresponding author: Yun Gao, MD, PhD

Department of Physiology, Basic Medical College of Nanchang University

461 Bayi Road, Nanchang, Jiangxi, China

Tel: (0086-791)86360586

Fax: (0086-791)86360586

Email: gaoyun@ncu.edu.cn
